# Integrating serum globulin into the ICF framework: a novel multidimensional predictive model for 1-year mRS outcomes in acute ischemic stroke

**DOI:** 10.1186/s40001-026-04004-9

**Published:** 2026-02-05

**Authors:** Chunxun Xiao, Hong Zhang, Dongli Chen, Yuqi Xiu, Zhili Liu, Yanchun Wu

**Affiliations:** 1 The First Affiliated Hospital of Shantou University Medical College, Shantou, 515041 Guangdong China; 2 School of Nursing, Shantou University Medical College, Shantou, 515041 Guangdong China

**Keywords:** Acute ischemic stroke, Serum globulin, ICF, Prognostic model, Nomogram

## Abstract

**Background:**

The long-term functional prognosis after ischemic stroke (IS) plays a crucial role in rehabilitation planning, yet it remains challenging to predict in clinical practice. Existing prognostic models primarily focus on short-term outcomes and lack integration of multidimensional determinants. Although elevated serum globulin levels have been associated with acute neuroinflammation and short-term disability, their prognostic significance for 1-year functional outcomes within a comprehensive biopsychosocial framework has not yet been established. To address these gaps, this study aimed to develop and validate a multidimensional prognostic model that integrates serum globulin as a key inflammatory biomarker into the International Classification of Functioning, Disability and Health (ICF) framework of the World Health Organization (WHO), with the objective of predicting 1-year functional outcome in patients with acute ischemic stroke (AIS).

**Methods:**

This prospective study consecutively enrolled 1,562 AIS patients at a Grade A tertiary hospital from 2021 to 2023; after data cleaning and screening, 1,356 cases were included for analysis. Baseline data were collected within 1 week of hospital admission. The study cohort was randomly divided into a training set (70%, *n* = 949) for model development and a validation set (30%, *n* = 407) for internal validation. The primary outcome was the patients’ functional status assessed using the modified Rankin Scale (mRS), 1-year post-admission. Predictors significant (*p* < 0.05) in univariate analysis within the training set were entered into backward stepwise multivariable logistic regression. The performance of the model was comprehensively evaluated using the area under the curve (AUC), Hosmer–Lemeshow test, calibration curve, and decision curve analysis (DCA).

**Results:**

Multivariable analysis identified six independent predictors (all *p* < 0.05): age, occupational status, BI, serum globulin, number of stroke episodes and NIHSS. A nomogram incorporating these predictors demonstrated excellent discrimination in both training (AUC = 0.90, 95% CI 0.88–0.93) and validation sets (AUC = 0.85, 95% CI 0.80–0.89). Calibration was good, with predicted probabilities (training: 22.23%; validation: 21.72%) closely matching the observed incidence of 22.20%, nonsignificant Hosmer–Lemeshow test results (*p* > 0.05), and well-aligned calibration curves. DCA confirmed the model’s superior net benefit over “treat-all” and “treat-none” strategies across clinically relevant high-risk thresholds (20–80%) in both training and validation cohorts.

**Conclusions:**

This study successfully integrated serum globulin into the ICF framework and constructed a prognostic model for the 1-year prognosis after AIS. It enables early identification of high-risk individuals and personalized rehabilitation strategies to improve long-term recovery.

**Supplementary Information:**

The online version contains supplementary material available at 10.1186/s40001-026-04004-9.

## Introduction

Acute ischemic stroke (AIS), accounting for 87% of all stroke cases [1], results from cerebral vessel occlusion leading to tissue ischemia and necrosis [2]. As a major global public health challenge, AIS is marked by high rates of incidence, mortality, disability, and recurrence [3]. According to the Global Burden of Disease Study, ischemic stroke (IS) resulted in approximately 7.8 million new cases, 3.59 million deaths, and 70.36 million disability-adjusted life years worldwide in 2021 [4]. China has the highest national incidence [5]. Although advancements in acute-phase treatment have improved survival rates, the majority of survivors experience persistent disability, imposing a substantial burden on families and society [6–8]. Therefore, accurate prognosis evaluation is essential for tailoring rehabilitation strategies and resource allocation. Existing evidence establishes age, gender, and the National Institutes of Health Stroke Scale (NIHSS) as reliable predictors of short-term prognosis [9–11]. However, most research has primarily concentrated on short-term outcomes [10, 12, 13], despite the fact that the consequences of stroke often persist well beyond the acute phase and post extended challenges for patients [14].

Serum globulin, a key indicator of the systemic inflammatory response, has emerged as a promising biomarker for its correlation with IS prognosis. Preclinical studies indicate that hyperglobulinaemia may exacerbate brain injury by promoting neuroinflammatory processes [15]. Clinical evidence further demonstrates that serum globulin levels measured 7-day post-intravenous thrombolysis are significantly and positively associated with the risk of mortality or severe disability at 3 months [16]. However, the independent predictive value of this biomarker for long-term functional outcomes requires confirmation through large-scale prospective studies.

The long-term prognosis of stroke survivors is frequently complicated by incomplete functional recovery, secondary complications, and psychosocial factors [17]. Identifying key determinants influencing long-term outcomes is crucial for accurate prognostic assessment. Such predictive capabilities would enable clinicians to more effectively plan rehabilitation strategies, prevent complications, and initiate early interventions for high-risk patients. However, research on long-term functional prognosis, particularly at the 1-year post-stroke timepoint, remains limited, and the prognostic significance of inflammatory biomarkers such as serum globulin has yet to be fully established.

Prediction models have gained increasing application in clinical practice. These models are designed to forecast future health outcomes integrating baseline predictors, thereby supporting clinical decision-making and improving patient prognosis [18]. They play a vital role in both disease diagnosis and prognosis prediction. However, a critical limitation of conventional AIS prognostic models lies in their primary dependence on biomedical parameters (e.g., imaging findings and comorbidities), frequently neglecting multidimensional factors that are crucial to long-term recovery, such as activity limitations and environmental factors [19].

To address these gaps, this study integrates serum globulin as a key inflammatory biomarker into the International Classification of Functioning, Disability and Health (ICF) framework of the comprehensive World Health Organization (WHO). The ICF adopts a biopsychosocial model, conceptualizing health outcomes through dynamic interactions among body functions and structures, activities and participation, environmental factors and personal factors [19]. This study aims to develop and validate a novel multidimensional prognostic model for predicting 1-year outcome as measured by the modified Rankin Scale (mRS) in AIS patients. This approach integrates a core inflammatory biomarker with a comprehensive perspective on post-stroke functioning, thereby enabling the early identification of high-risk individuals and facilitating personalized rehabilitation planning to improve long-term outcomes.

## Methods

### Study population

This prospective cohort study consecutively enrolled patients with AIS through convenience sampling at a Grade A tertiary hospital in Guangdong Province, China, between January 2021 and December 2023. Participants who met the predefined inclusion and exclusion criteria, were identified and recruited within 1 week of hospital admission.

### Eligibility criteria

Patients were included if they met the following criteria: (1) diagnosed with ischemic stroke according to the 2018 Chinese Guidelines for the Diagnosis and Treatment of Acute Ischemic Stroke [20], confirmed by cranial CT or MRI; (2) aged ≥ 18 years; (3) within 1 week after acute ischemic stroke onset; and (4) conscious and capacity for reliable communication, with access to devices necessary for scheduled follow-up. Patients were excluded if they met the following conditions: (1) comorbid malignancies, end-stage failure of major organs (heart, lung, liver, or kidney), or other severe non-stroke conditions predicted to substantially reduce life expectancy or independently cause functional disability; (2) pre-existing neurological disorders or a history of stroke leading to severe residual functional impairment; or (3) severe aphasia, psychiatric disorders, or cognitive impairment significantly impeding reliable information provision. Participants were considered lost to follow-up: (1) contact could not be established after two daily phone attempts for three consecutive days or (2) they voluntarily withdrew or explicitly refused continued participation during the follow-up period.

### Baseline assessments

A comprehensive set of variables was systematically collected via face-to-face patient interviews and electronic medical record systems at baseline in accordance with the ICF framework (Fig. S1), covering the following four domains:

1. Personal factors: age, gender, marital status, smoking, drinking and family history.

2. Environmental factors: educational level, occupational status and caregiver.

3. Activities and participation: Barthel Index (BI).

4. Body functions:

(1) Laboratory parameters:

Hematological parameters: white blood cell (WBC), red blood cell (RBC), hemoglobin (Hb), platelet (PLT), neutrophil (NEU), lymphocyte (LYM) and monocyte (MONO).

Hepatic function: total protein (TP), serum albumin, serum globulin, alanine transaminase (ALT) and aspartate transaminase (AST).

Metabolic parameters: glucose (GLU), total cholesterol (TC), triglycerides (TG), high-density lipoprotein cholesterol (HDL) and low-density lipoprotein cholesterol (LDL).

(2) Comorbidities: hypertension, diabetes mellitus, atrial fibrillation and hyperlipidaemia.

(3) Stroke severity indicators: number of stroke episodes and National Institutes of Health Stroke Scale (NIHSS).

### Activities of daily living assessment

The BI was originally developed by Mahoney and Barthel in 1965 [21]. It is a validated 10-item scale that quantifies independence in basic activities of daily living, including feeding, bathing, grooming, dressing, bowel and bladder control, toileting, transfers, ambulation (45 m) and stair climbing. The assessment employs a weighted scoring system, ranging from 0 to 15 points, which is based on the level of assistance required. Total scores range from 0 (complete dependence) to 100 (full functional autonomy), with higher scores indicating greater independence [22].

### Stroke severity assessment

The NIHSS was used to measure the severity of neurological impairment in stroke patients [23]. This 11-item scale, developed by the National Institutes of Health, is a globally validated instrument that systematically evaluates consciousness, gaze, visual fields, facial palsy, motor function (upper and lower limbs), limb ataxia, sensory perception, language, dysarthria and extinction/inattention [24]. Each item is scored on an ordinal scale of 0–2 or 0–4, with cumulative scores ranging from 0 (no deficits) to 42 (maximum deficits); higher scores correlate with greater neurological impairment [25].

### Outcome assessment at 1-year follow-up

Prognosis was assessed by structured telephone interviews using mRS conducted by centrally trained investigators at 1-year post-stroke. The mRS, originally developed by Dr John Rankin in 1957 and later modified by Charles Warlow et al. in the late 1980 s [26], is a widely adopted single-item global disability scale in stroke outcome research that quantifies functional dependence following neurological events [27, 28]. Scores range from 0 (asymptomatic) to 6 (fatal outcome). Functional outcomes were categorized as favorable (mRS ≤ 2) vs. unfavorable (mRS > 2). Telephone assessment has been shown to demonstrate good inter-rater agreement with in-person assessment, with a weighted kappa of 0.71 (95% CI 0.59–0.82) [29].

### Sample size calculation

This study employed the events per variable (EPV) principle for sample size calculation, requiring at least 10 events per variable [30]. With an anticipated final number of predictors ranging from 10 to 15, and based on previous research indicating a prevalence of unfavorable 1-year prognosis in AIS patients of approximately 25.8% [31], a loss to follow-up rate of 15–20% was also considered. The calculated required sample size ranges from 457 to 726 cases. Final enrollment (*n* = 1356) substantially exceeded this requirement, ensuring adequate statistical power.

### Statistical analysis

Analyses were conducted using R version 4.4.3 (R Foundation for Statistical Computing, Vienna, Austria) with the following packages employed: caret for data partitioning, stats and ggplot2 for normality assessment and visualization, MASS and car for regression modeling, rms for nomogram construction, pROC and ResourceSelection for model evaluation, and rmda for decision curve analysis. Continuous variables underwent normality assessment through Shapiro–Wilk testing complemented by visual inspection of Q–Q plots. Normally distributed variables are expressed as mean ± standard deviation (SD) and compared using independent *t* tests, while non-normally distributed variables are presented as median (interquartile range, IQR) and analyzed with Mann–Whitney *U* tests. Categorical variables are reported as counts (percentages) and compared using chi-square tests or Fisher’s exact test. The study cohort (*n* = 1356) was randomly divided into a training set (70%, *n* = 949) and a validation set (30%, *n* = 407). This random partitioning was performed to mitigate selection bias and ensure the comparability of the training and validation sets. The training set was utilized for model development, including variable selection and parameter estimation, while the validation set was reserved for providing an unbiased assessment of the final model’s performance and generalizability. Within the training set, variables that demonstrated significant in univariable analyses (*p* < 0.05) were subsequently entered into the multivariable logistic regression model employing backward stepwise elimination (removal threshold *p* ≥ 0.05). Multicollinearity among variables was evaluated using variance inflation factors (VIF). Statistically significant predictors retained in the final model (*p* < 0.05) informed the construction of a nomogram based on regression coefficients. Model discrimination was quantified using the area under the receiver operating characteristic curve (AUC), while calibration was assessed through Hosmer–Lemeshow goodness-of-fit testing (*p* > 0.05 indicating adequate calibration) supplemented by calibration plots comparing predicted vs. observed probabilities. Clinical utility across decision thresholds was evaluated using decision curve analysis. A statistically significant level was set at *p* < 0.05 (two-tailed).

## Results

### Baseline characteristics

**Fig. 1:**
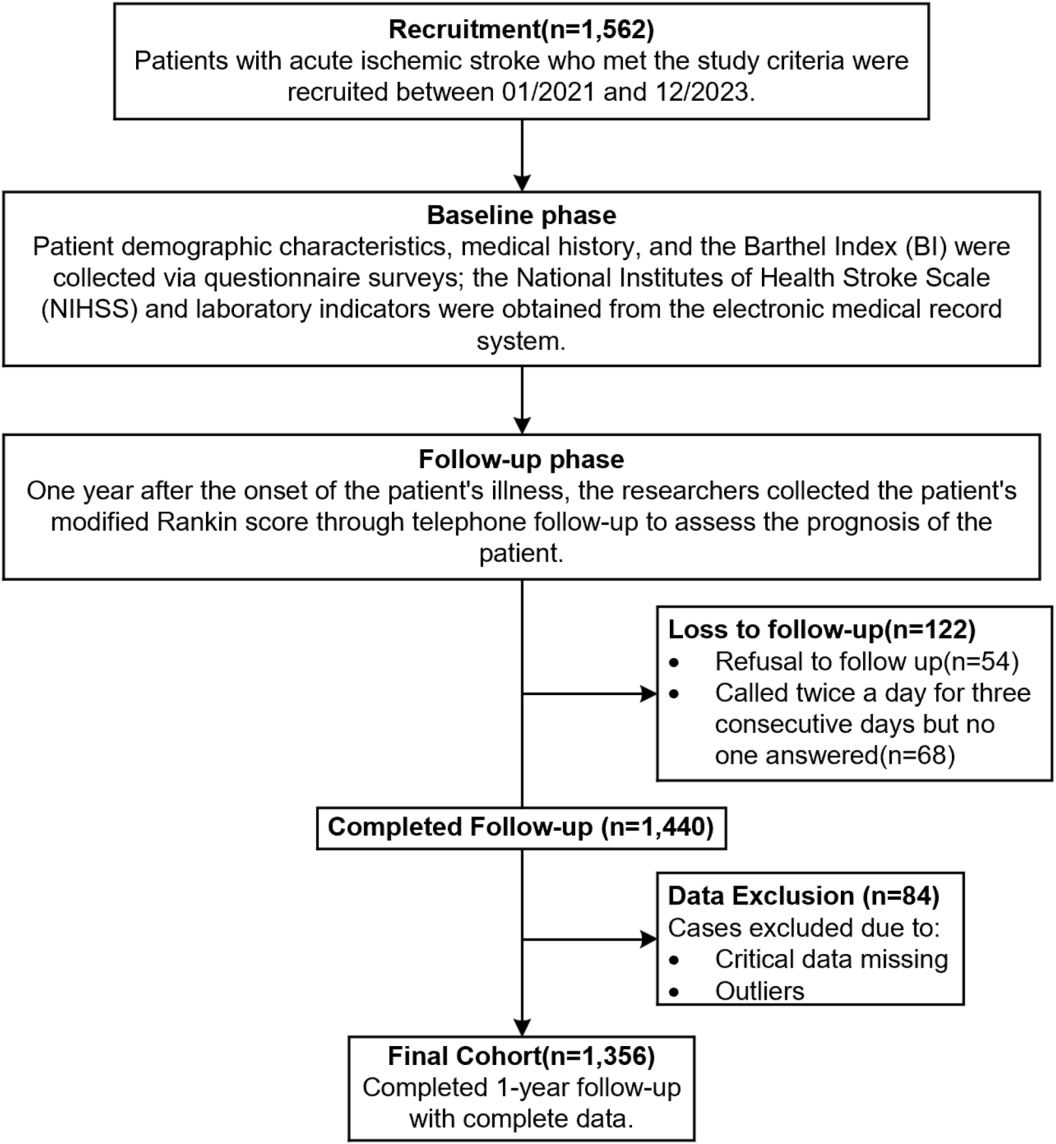
Flowchart of patient screening in the acute ischemic stroke prospective cohort study

A total of 1,562 participants were initially enrolled. During the follow-up period, 122 participants were withdrawn (54 declined further participation; 68 were lost to follow-up) and 84 were excluded due to critically missing data or outlier values. Consequently, 1356 participants were included in the final analysis, resulting in a cohort retention rate of 86.8% (1356/1562). All participants included in the analysis completed the 1-year follow-up with comprehensive data records. The participant screening process is detailed in Fig. 1.

Participant characteristics were well-balanced between the training set (70%, *n* = 949) and validation set (30%, *n* = 407) (all between-group comparisons *p* > 0.05; Table S1). VIF values for all variables were < 10, indicating absence of significant multicollinearity. Shapiro–Wilk testing revealed non-normal distributions for all continuous variables (*p* < 0.05). Subsequent analyses were thus performed using non-parametric methodologies.

This cohort study comprised 1356 AIS patients, of whom 1055 (77.80%) exhibited favourable prognosis and 301 (22.20%) had unfavourable prognosis. The median age was 66.00 (IQR: 58.00–73.00) years, with males constituting 62.24% (*n* = 844). Most participants (97.20%, *n* = 1,318) were married. Lifestyle characteristics indicated 55.68% (*n* = 755) were non-smokers and 85.10% (*n* = 1,154) were non-drinkers. Educational level distribution was: illiterate (19.69%, *n* = 267), primary school (41.08%, *n* = 557), junior high school (22.42%, *n* = 304), and high school or above (16.81%, *n* = 228). Occupational status included: retired (22.35%, *n* = 303), employed (11.65%, *n* = 158), unemployed (64.23%, *n* = 871) and other (1.77%, *n* = 24). Hypertension (78.76%, *n* = 1068) and diabetes mellitus (41.00%, *n* = 556) were the most prevalent comorbidities. First-ever stroke accounted for 80.16% (*n* = 1087) of cases, while 19.84% (*n* = 269) had recurrent stroke history (≥ 2 episodes). Other data are shown in Table S1.

Table 1 presents the baseline characteristics of the training set and presents the results of univariate analysis comparing clinical features between patients with favourable and unfavourable prognosis. Significant intergroup differences emerged for: age, gender, smoking, drinking, family history, occupational status, caregiver, BI, WBC, RBC, Hb, NEU, LYM, MONO, serum albumin, serum globulin, AST, GLU, LDL, diabetes mellitus, atrial fibrillation, number of stroke episodes and NIHSS.

**Table 1:** Univariate analysis of factors associated with 1-year prognosis

Variables	Overall (*N* = 949)	Favorable prognosis group (*n* = 747)	Unfavorable prognosis group (*n* = 202)	Z/χ^2^	*P*
Age, years	66.00 (58.00, 73.00)	65.00 (57.00,72.00)	71.50 (66.00, 78.00)	− 8.55	< 0.001
Gender, *n*(%)				15.99	< 0.001
Male	585 (61.64)	485 (64.93)	100 (49.50)		
Female	364 (38.36)	262 (35.07)	102 (50.50)		
Marital status, *n*(%)				0.00	1.000
Unmarried	23 (2.42)	18 (2.41)	5 (2.48)		
Married	926 (97.58)	729 (97.59)	197 (97.52)		
Smoking, *n*(%)				11.05	< 0.001
No	527 (55.53)	394 (52.74)	133 (65.84)		
Yes	422 (44.47)	353 (47.26)	69 (34.16)		
Drinking, *n*(%)				5.45	0.020
No	811 (85.46)	628 (84.07)	183 (90.59)		
Yes	138 (14.54)	119 (15.93)	19 (9.41)		
Family history, *n*(%)				4.08	0.043
No	846 (89.15)	658 (88.09)	188 (93.07)		
Yes	103 (10.85)	89 (11.91)	14 (6.93)		
Educational level, *n*(%)				7.18	0.066
Illiterate	196 (20.65)	143 (19.14)	53 (26.24)		
Primary school	387 (40.78)	303 (40.56)	84 (41.58)		
Junior high school	209 (22.02)	169 (22.62)	40 (19.80)		
High school or above	157 (16.54)	132 (17.67)	25 (12.38)		
Occupational status, *n*(%)				28.91	< 0.001
Retired	216 (22.76)	159 (21.29)	57 (28.22)		
Employed	110 (11.59)	107 (14.32)	3 (1.49)		
Unemployed	609 (64.17)	468 (62.65)	141 (69.80)		
Other	14 (1.48)	13 (1.74)	1 (0.50)		
Caregiver, *n*(%)				16.83	< 0.001
Spouse	355 (37.41)	288 (38.55)	67 (33.17)		
Children	496 (52.27)	369 (49.40)	127 (62.87)		
Self	98 (10.33)	90 (12.05)	8 (3.96)		
BI	90.00 (50.00, 100.00)	100.00 (75.00, 100.00)	25.00 (0.00, 55.00)	− 16.87	< 0.001
WBC, × 10⁹/L	7.63 (6.32, 9.36)	7.48 (6.22, 9.06)	8.07 (6.92, 10.28)	− 3.95	< 0.001
RBC, × 10^12^/L	4.44 (4.12, 4.81)	4.48 (4.16, 4.84)	4.29 (3.95, 4.73)	− 3.90	< 0.001
Hb, g/L	132.00 (122.00, 143.00)	133.00 (123.00, 144.00)	129.00 (115.00, 139.00)	− 4.02	< 0.001
PLT, × 10⁹/L	233.00 (192.00, 273.00)	232.00 (193.50, 271.00)	234.50 (188.25, 276.75)	− 0.38	0.703
NEU, × 10⁹/L	5.11 (3.98, 6.97)	4.92 (3.88, 6.72)	5.75 (4.46, 8.11)	− 3.90	< 0.001
LYM, × 10⁹/L	1.73 (1.31, 2.29)	1.77 (1.37, 2.33)	1.53 (1.16, 2.02)	− 4.50	< 0.001
MONO, × 10⁹/L	0.52 (0.40, 0.70)	0.52 (0.40, 0.68)	0.58 (0.41, 0.77)	− 2.39	0.017
TP, g/L	65.39 (62.00, 69.00)	65.27 (61.90, 68.78)	65.92 (62.59, 69.41)	− 1.51	0.130
Serum albumin, g/L	38.31 (36.47, 40.68)	38.45 (36.77, 40.70)	37.64 (35.60, 40.18)	− 3.39	< 0.001
Serum globulin, g/L	27.14 (24.46, 29.88)	26.79 (24.07, 29.59)	28.43 (25.63, 31.22)	− 5.00	< 0.001
ALT, U/L	17.04 (12.92, 23.74)	17.20 (13.05, 24.11)	16.56 (12.34, 22.37)	− 1.52	0.128
AST, U/L	19.42 (16.04, 24.41)	19.14 (15.82, 23.84)	20.36 (17.21, 26.84)	− 3.04	0.002
GLU, mmol/L	5.55 (4.85, 7.42)	5.46 (4.79, 7.22)	6.17 (5.17, 7.87)	− 3.94	< 0.001
TC, mmol/L	4.75 (3.99, 5.51)	4.78 (4.04, 5.50)	4.68 (3.82, 5.57)	− 1.25	0.210
TG, mmol/L	1.12 (0.92, 1.36)	1.11 (0.92, 1.36)	1.12 (0.94, 1.35)	− 0.49	0.624
HDL, mmol/L	2.57 (1.28, 3.35)	2.62 (1.25, 3.35)	2.42 (1.39, 3.27)	− 0.80	0.423
LDL, mmol/L	1.58 (1.09, 2.56)	1.66 (1.11, 2.60)	1.49 (0.98, 2.41)	− 2.21	0.027
Hypertension, *n*(%)				2.28	0.131
No	206 (21.71)	170 (22.76)	36 (17.82)		
Yes	743 (78.29)	577 (77.24)	166 (82.18)		
Diabetes Mellitus, *n*(%)				4.21	0.040
No	567 (59.75)	459 (61.45)	108 (53.47)		
Yes	382 (40.25)	288 (38.55)	94 (46.53)		
Atrial Fibrillation, *n*(%)				12.94	< 0.001
No	895 (94.31)	715 (95.72)	180 (89.11)		
Yes	54 (5.69)	32 (4.28)	22 (10.89)		
Hyperlipidemia, *n*(%)				2.23	0.135
No	820 (86.41)	639 (85.54)	181 (89.60)		
Yes	129 (13.59)	108 (14.46)	21 (10.40)		
Number of stroke episodes, *n*(%)				31.84	< 0.001
Once	763 (80.40)	628 (84.07)	135 (66.83)		
Twice	155 (16.33)	102 (13.65)	53 (26.24)		
≥ 3 times	31 (3.27)	17 (2.28)	14 (6.93)		
NIHSS	3.00 (1.00, 5.00)	2.00 (1.00, 4.00)	7.00 (3.00,10.00)	− 11.84	< 0.001

Data presented are median (Q1, Q3) or *n* (%)

BI, Barthel Index; WBC, white blood cell; RBC, red blood cell; Hb, hemoglobin; PLT, platelet; NEU, neutrophil; LYM, lymphocyte; MONO, monocyte; TP, total protein; ALT, alanine transaminase; AST, aspartate transaminase; GLU, glucose; TC, total cholesterol; TG, triglycerides; HDL, high density lipoprotein; LDL, low density lipoprotein; NIHSS, National Institutes of Health Stroke Scale

Variables exhibiting statistical significance in univariate analysis were incorporated into a multivariate logistic regression model (Table 2). The analysis identified the following independent predictors of unfavorable 1-year prognosis in AIS patients (*p* < 0.05): age (OR: 1.03, 95% CI 1.01–1.05, *p* = 0.017), occupational status (vs. retired: employed: OR: 0.18, 95% CI 0.04–0.76, *p* = 0.020; unemployed: OR: 0.54, 95% CI 0.32–0.90, *p* = 0.019), BI (OR: 0.96, 95% CI 0.95–0.96, *p* < 0.001), serum globulin (OR: 1.06, 95% CI 1.01–1.12, *p* = 0.012), number of stroke episodes (vs. once: twice: OR: 2.32, 95% CI 1.37–3.94, *p* = 0.002; ≥ 3 times: OR: 2.61, 95% CI 0.92–7.38, *p* = 0.071), and NIHSS (OR: 1.08, 95% CI 1.02–1.15, *p* = 0.007). These predictors were subsequently used to construct a nomogram for 1-year prognosis prediction.

**Table 2:** Independent predictors of 1-year prognosis identified by multivariate logistic regression analysis in the training set

Variables	β	S.E	Z	*P*	OR (95% CI)
Intercept	− 2.19	1.19	− 1.85	0.065	0.11 (0.01 ~ 1.14)
Age	0.03	0.01	2.38	0.017	1.03 (1.01 ~ 1.05)
Occupational status					
Retired					1.00 (Reference)
Employed	− 1.73	0.74	− 2.33	0.020	0.18 (0.04 ~ 0.76)
Unemployed	− 0.62	0.26	− 2.34	0.019	0.54 (0.32 ~ 0.90)
Other	− 1.39	1.23	− 1.13	0.259	0.25 (0.02 ~ 2.78)
BI	− 0.04	0.00	− 10.68	< 0.001	0.96 (0.95 ~ 0.96)
Serum globulin	0.06	0.02	2.50	0.012	1.06 (1.01 ~ 1.12)
Number of stroke episodes, *n*(%)					
Once					1.00 (Reference)
Twice	0.84	0.27	3.12	0.002	2.32 (1.37 ~ 3.94)
≥ 3 times	0.96	0.53	1.81	0.071	2.61 (0.92 ~ 7.38)
NIHSS	0.08	0.03	2.68	0.007	1.08 (1.02 ~ 1.15)

*OR* Odds Ratio, *CI* Confidence Interval

### Development of a prediction model

**Fig. 2:**
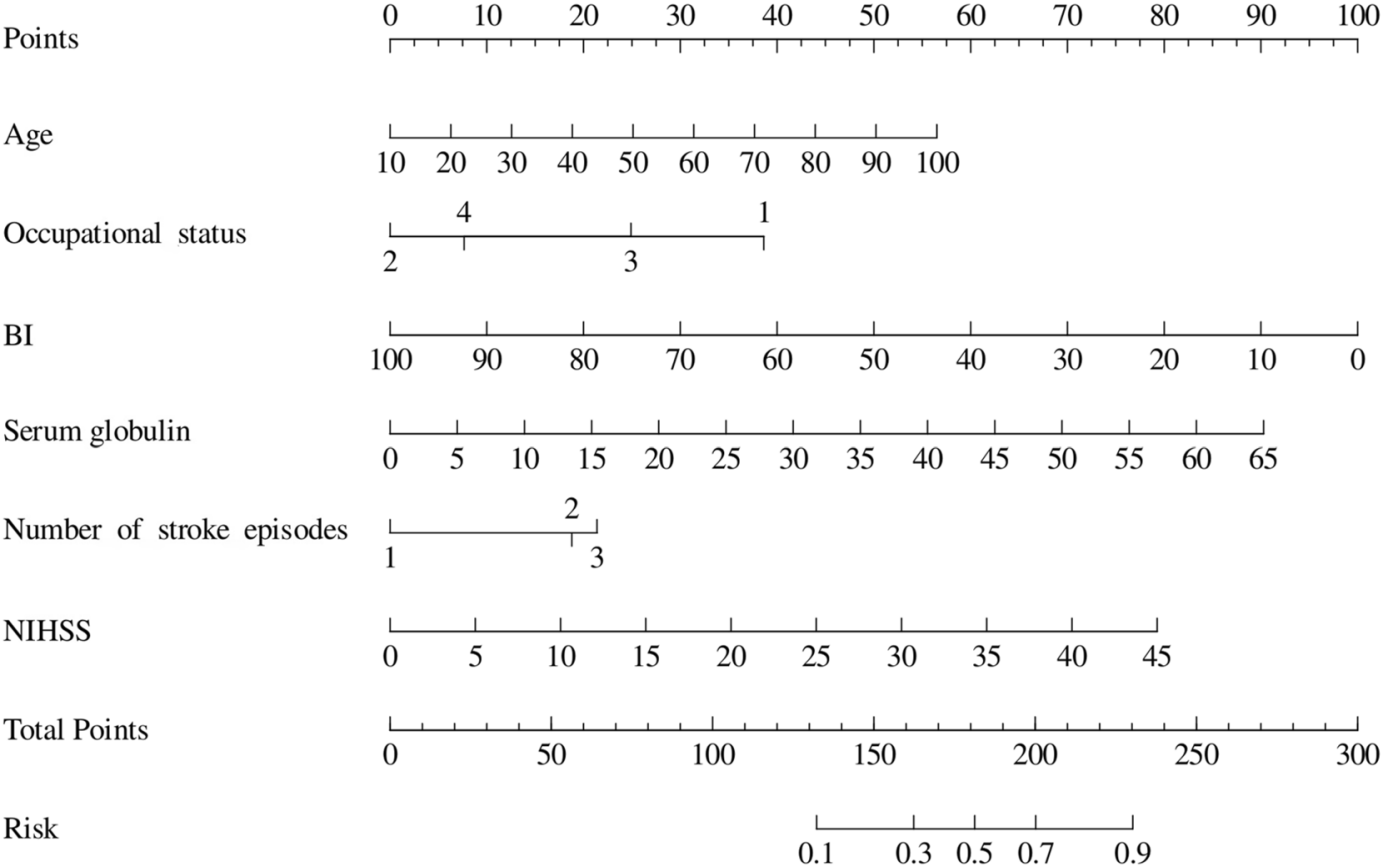
Nomogram for predicting the 1-year prognosis of patients with acute ischemic stroke

Based on the six aforementioned independent predictors, a nomogram was constructed to predict 1-year prognosis in AIS patients (Fig. 2). This visual model integrates all predictors by enabling users to plot individual variable values onto a points scale. The total points are then mapped to the probability scale at the bottom, directly yielding the estimated risk probability (range: 0.1–0.9).

### Model performance and validation

#### Discrimination performance

**Fig. 3:**
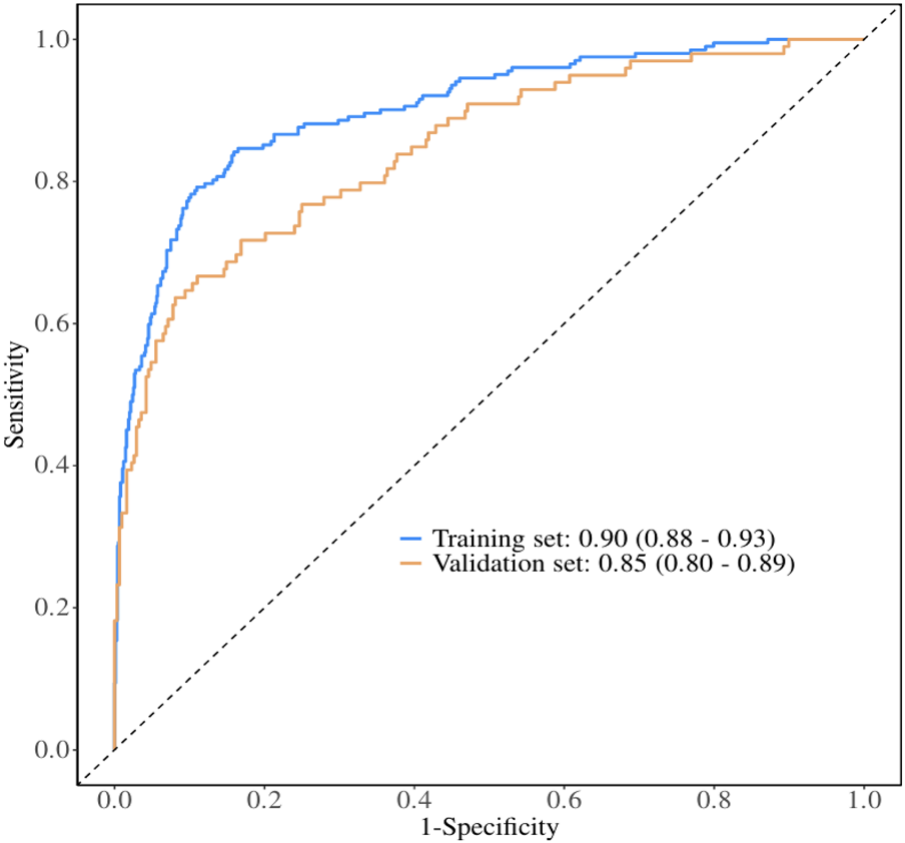
Receiver operating characteristic curves of the prediction model

The model demonstrated strong discriminative ability (Fig. 3), achieving an AUC of 0.90 (95% CI 0.88–0.93) in the training set and 0.85 (95% CI 0.80–0.89) in the validation set. Both AUC values significantly outperformed that of a random classifier (AUC = 0.5; dashed diagonal line). Although a 5% AUC reduction was observed during validation, performance remained excellent (AUC > 0.80). Additional classification metrics (Table S2) indicated expected generalization patterns: validation sensitivities (0.84 vs. training 0.89), specificities (0.69 vs. 0.79), and accuracies (0.80 vs. 0.87) decreased within clinically acceptable ranges. Notably, the validated model maintained a high positive predictive value (PPV = 0.89) despite a lower negative predictive value (NPV = 0.58), highlighting its increased utility for high-risk patient stratification.

#### Calibration performance

**Fig. 4:**
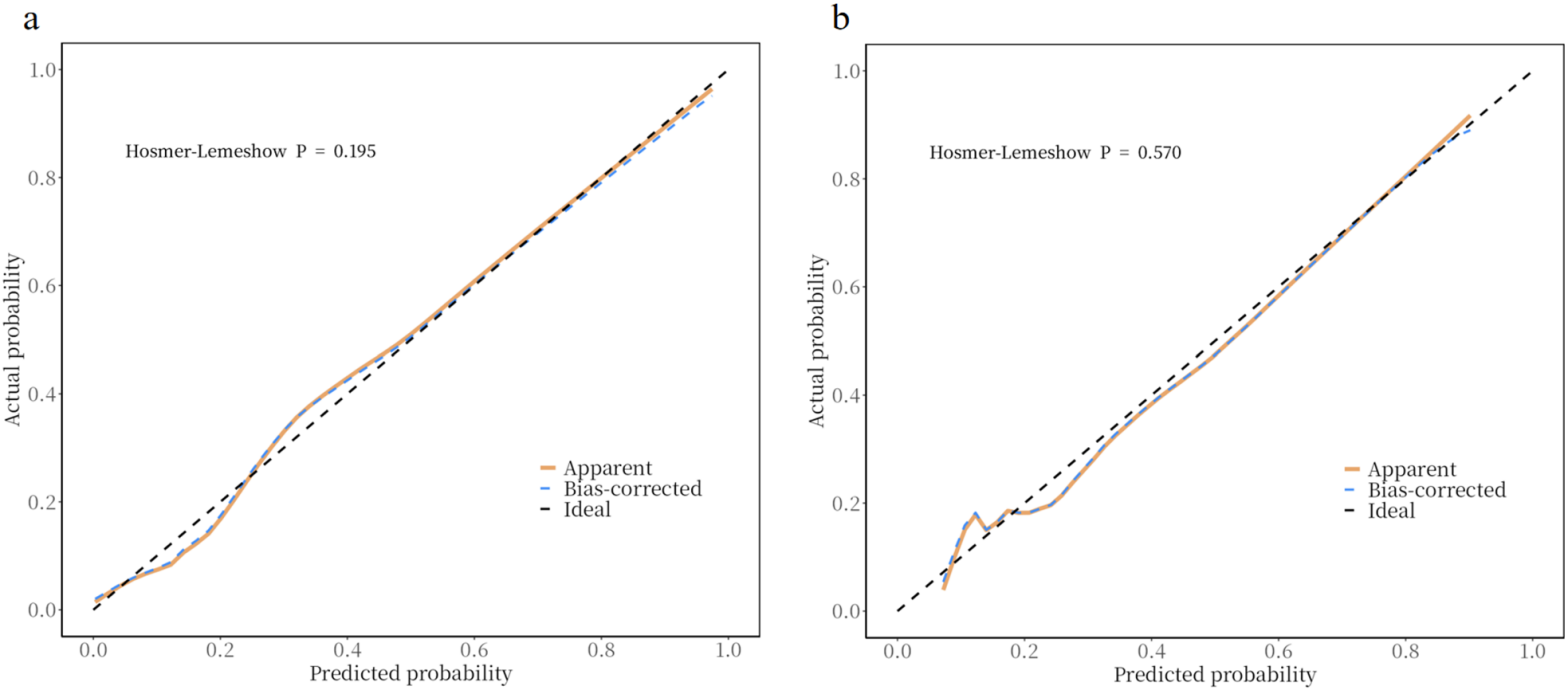
Calibration curves of the prediction model. **a** Training Set **b** Validation Set

The model’s mean predicted probability of unfavorable prognosis was 22.23% in the training set and 21.72% in the validation set. Quantitative assessment via Hosmer–Lemeshow tests demonstrated excellent calibration in both the training (χ^2^ = 11.12, df = 8, *p* = 0.195) and validation sets (χ^2^ = 6.69, df = 8, *p* = 0.570). Calibration curves provided complementary visual evidence, with bootstrap-corrected lines demonstrating close agreement with ideal reference lines in both the training and validation sets (Fig. 4). The higher validation *p* value (0.570) relative to training (0.195) indicates superior calibration stability in external data sets.

### Clinical decision utility

**Fig. 5:**
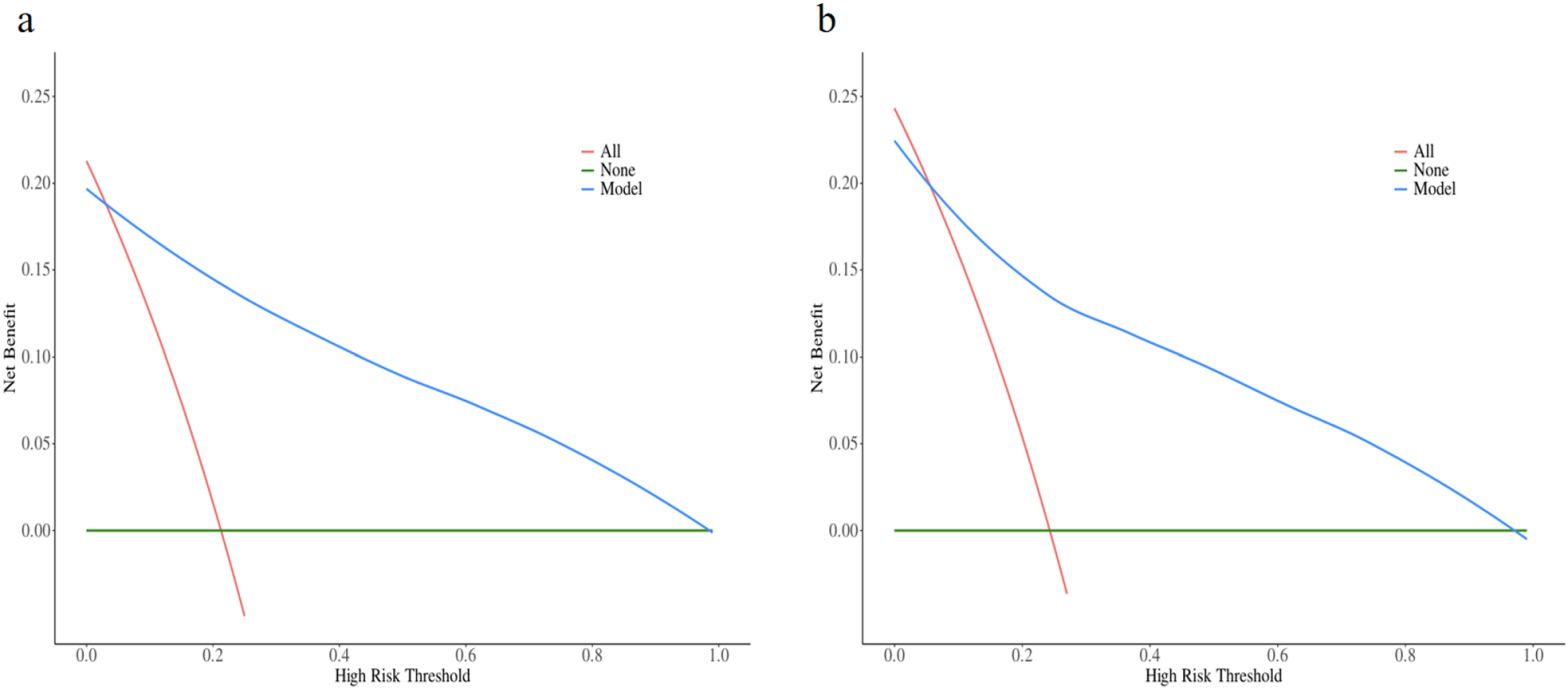
Decision curve analysis of the prediction model. **a** Training Set **b** Validation Set

The decision curve analysis (DCA) was performed to evaluate the clinical utility of the predictive model across a range of high-risk thresholds, comparing outcomes in both the training and validation sets (Fig. 5). As illustrated in Fig. 5, the net benefit curves demonstrate the performance of the Model (blue), alongside the strategies of treating all patients (red) and treating none (green). Across both cohorts (a: training set; b: validation set), the Model’s net benefit curve remained consistently above both the ‘All’ and ‘None’ curves over a broad range of clinically relevant risk thresholds (approximately 0–0.8). As the high-risk threshold increased, the net benefit of the Model declined gradually. Notably, the ‘All’ curve exhibited a steeper decline than the Model, while the ‘None’ curve remained at zero net benefit throughout, serving as a baseline reference. The close agreement in the shape and relative positions of the curves between the training and validation sets indicates that the model provides robust and generalizable clinical utility, offering superior net benefit compared to default strategies for intervention decisions across most threshold probabilities.

## Discussion

This study represents the first effort to develop and validate a multidimensional nomogram model for predicting 1-year prognosis in patients with acute ischemic stroke, grounded in the ICF theoretical framework. The model integrates serum globulin into the ICF framework. It demonstrated robust discriminative ability, adequate calibration, and substantial clinical utility in both training and validation sets. Crucially, the model’s predictive accuracy was further underscored by the close numerical alignment between the predicted probability of an unfavorable prognosis (21.72%) in the validation set and the actual observed incidence (22.20%). These results provide strong evidence for the model’s applicability in individualised risk stratification and clinical decision-making. The final multivariable model identified six independent predictors, all of which demonstrated biologically plausible associations with stroke pathophysiology and recovery mechanisms.

In the training set, patients in the unfavorable prognosis group were significantly older than those in the favorable prognosis group (median age: 71.50 vs. 65.00 years, *p* < 0.001), consistent with age-related declines in physiological reserve, increased comorbidities, and attenuated neuroplasticity [32]**.** The median NIHSS in the training set was 3.00 (IQR: 1.00–5.00), yet significantly higher scores were observed in the unfavorable prognosis group (median: 7.00 vs. 2.00; *p* < 0.001), aligning with previous literature [33]. Age [10, 34] and NIHSS [33–36] are well-established predictors of unfavorable prognosis. The 90-day mRS score remains the most widely used outcome measure in current research [10, 33, 36–38]. However, limited evidence is available on the long-term prognosis in AIS patients beyond 1 year. This study confirms the predictive validity of these factors for long-term (1-year) prognosis.

This study demonstrates that occupational status remains a significantly independent predictor of prognosis at 1 year after AIS, even following adjustment for conventional factors, including age, BI, serum globulin, number of stroke episodes and NIHSS. Employed group exhibited an 82% lower risk of unfavorable prognosis compared to retired group (OR = 0.18, *p* = 0.020). This observed association may be attributed to socioeconomic advantages inherent to employment, including stable income, comprehensive healthcare, sustained social engagement, and psychological purpose—factors that may collectively facilitate better access to rehabilitation and improved adherence to treatment protocols [39–41]. Notably, although the unemployed group showed a lower risk compared to the retired group (OR = 0.54, *p* = 0.019), their risk remained higher than that of the employed group. Unemployed group may still maintain the motivation to re-enter the workforce, but frequently face socioeconomic challenges, such as income loss, insurance instability, as well as psychological distress, and restricted access to rehabilitation services [42, 43]. Moreover, employability intrinsically indicates favorable premorbid health status and functional reserve. Furthermore, it is important to highlight that returning to work is increasingly acknowledged as a significant determinant of life satisfaction and well-being after stroke [44, 45]. Therefore, beyond its function as a prognostic indicator, proactive efforts to facilitate return to work, where both feasible and desired by the patient—should be prioritized within post-discharge support and rehabilitation programs specifically designed for working-age individuals.

Regarding number of stroke episodes, this study found that patients who have suffered two strokes exhibited a significantly higher risk of unfavorable prognosis compared to those with only one stroke (OR = 2.32, *p* = 0.002). This results consistent with previous research [46, 47], which have demonstrated that recurrent stroke increases mortality risk and negatively affects overall stroke prognosis. Patients with three or more strokes showed a comparable trend (OR = 2.61), although statistical significance was not achieved (*p* = 0.071). This may be due to limited statistical power resulting from the relative small sample size in the ≥ 3 strokes group (3.10%, *n* = 42), potentially masking a true effect. Furthermore, patients with multi-stroke may exhibit greater heterogeneity in locations of infarctions, comorbidity burden, and cumulative deficits. This heterogeneity likely exacerbated outcome variability and contributed to this non-significant result. Nevertheless, the substantial point estimate (OR = 2.61), combined with the pronounced and statistically significant adverse effect observed after the second stroke, strongly indicates a consistent, clinically meaningful pattern of progressively increasing risk with each recurrent stroke event. Therefore, heightened clinical vigilance, comprehensive management, and intensive secondary prevention measures should be clinically implemented for this high-risk patients population. Future larger-scale studies involving expanded cohorts are warranted to validate the magnitude of this risk association and determine its clinical relevance.

This study demonstrates that the BI remains a significant independent predictor of prognosis at 1 year after AIS, with each one-point increase in BI corresponding to approximately a 4% reduction in the risk of an unfavorable prognosis (OR = 0.96, *p* < 0.001). This suggests that improving from dependence levels (e.g., 30 points) to minimal assistance levels (e.g., 60–70 points) would result in a significant risk reduction of over 40%, highlighting the substantial clinical protective effect. These findings robustly corroborate previous research indicating that favourable functional reserve powerfully safeguards long-term neurological recovery [48, 49]. Patients maintaining or rapidly regaining fundamental self-care abilities exhibit enhanced neurological resilience post-injury, thereby facilitating the activation of effective compensatory and restorative mechanisms [8].

This study demonstrates that serum globulin is an independent predictor of 1-year outcomes in AIS patients. Each unit increase in serum globulin independently predicted elevated risk (OR = 1.06, *p* = 0.012), solidifying its value as an accessible prognostic biomarker. Previous studies linked elevated globulin to short-term outcomes in mild acute ischemic stroke (e.g., mortality, discharge disposition [50]) or thrombolysis cohorts [51]. Our prospective data extend this relationship to 1-year disability trajectories, suggesting that globulin-reflected pathophysiology persists beyond acute hospitalization. Chronic inflammation or immune dysregulation—indicated by elevated globulin—may perpetuate neural injury and impede recovery [39, 52]. As a low-cost, routine biomarker, globulin adds a biologically actionable dimension to prognostic models, implying that subclinical inflammation independently influences rehabilitation.

## Conclusion

In summary, our predictive model integrating serum globulin within the ICF framework provides a reliable tool for predicting 1-year functional outcome in patients with acute ischemic stroke. This model enables early identification of high-risk individuals and supports personalized rehabilitation planning, thereby contributing to improved long-term recovery after stroke.

### Strengths

This study incorporated the biopsychosocial model through the ICF framework, ensuring that the selected variables covered relevant health dimensions and avoided the limitations of focusing on a single aspect. The predictive factors in this model are routinely assessed and objectively measured clinical parameters, enhancing its feasibility for implementation in diverse clinical settings. Furthermore, the study’s prospective design minimizes recall bias, while the large patient cohort provides robust statistical power and enhances generalizability. Outcomes assessment at the 1-year follow-up effectively captures meaningful changes relevant to chronic conditions, thereby ensuring robust data reliability and model stability over a clinically significant timeframe.

### Limitations and future directions

This study has several limitations. First, it was conducted at a single centre with only internal validation, lacking external validation on independent cohorts. This limits the generalisability of the findings, so future research should consider multi-centre collaborations or utilise external data sets to confirm the robustness and clinical applicability of the models. Second, the enrolled patient population predominantly comprised individuals with mild-to-moderate stroke severity, as reflected by the low median NIHSS. This potentially restricts the applicability of our conclusions to patients with severe strokes. Future studies should specifically target and analyse patients with high NIHSS scores and under-represented stroke subtypes to broaden the scope and validity of the findings. Third, it did not cover all components of the ICF framework. For instance, although environmental factors in the model represent significant determinants of community participation, direct assessment of participation outcomes at the community level—such as return to work or social roles—was not conducted. In addition, aspects of body structure, including detailed neuroimaging characteristics, were not incorporated. Future research could be strengthened by employing specialized participation measures such as the Participation Assessment with Recombined Tools-Objective (PART-O) and integrating neuroimaging data to develop more comprehensive models.

## Supplementary Information

Supplementary Material 1

## Data Availability

The datasets used and/or analysed during the current study are available from the corresponding author on reasonable request.

## Abbreviations

AIS: Acute ischemic stroke
AUC: Area under the curve
BI: Barthel Index
CI: Confidence interval
DCA: Decision curve analysis
ICF: International Classification of Functioning, Disability and Health
IS: Ischemic stroke
mRS: Modified Rankin Scale
NIHSS: National Institutes of Health Stroke Scale
NPV: Negative predictive value
OR: Odds ratio
PPV: Positive predictive value
ROC: Receiver operating characteristic
VIF: Variance inflation factors
WHO: World Health Organization
