# Supplementary material for: Integrating serum globulin into the ICF framework: a novel multidimensional predictive model for 1-year mRS outcomes in acute ischemic stroke
Source: Eur J Med Res. 2026 Feb 5;31:387. doi: 10.1186/s40001-026-04004-9 (PMC12964722; doi:10.1186/s40001-026-04004-9)
Supplement: Supplementary file 1 — Supplementary Material 1 [file 40001_2026_4004_MOESM1_ESM.docx]

**Supplementary Information**

Table S1 Baseline characteristics in training set and validation set

| Variables | Overall  (N = 1,356) | Training Set  (n = 949) | Validation Set  (n = 407) | | Z/χ² | | *P* | |
| --- | --- | --- | --- | --- | --- | --- | --- | --- |
| Age, years | 66.00  (58.00, 73.00) | 66.00  (57.00, 72.50) | 66.00  (58.00, 73.00) | | -0.82 | | 0.410 | |
| Gender, n(%) |  |  |  | | 0.48 | | 0.488 | |
| Male | 844 (62.24) | 259 (63.64) | 585 (61.64) | |  | |  | |
| Female | 512 (37.76) | 148 (36.36) | 364 (38.36) | |  | |  | |
| Marital status, n(%) |  |  |  | | 1.67 | | 0.197 | |
| Unmarried​ | 38 (2.80) | 15 (3.69) | 23 (2.42) | |  | |  | |
| Married | 1,318 (97.20) | 392 (96.31) | 926 (97.58) | |  | |  | |
| Smoking, n(%) |  |  |  | | 0.03 | | 0.868 | |
| No | 755 (55.68) | 228 (56.02) | 527 (55.53) | |  | |  | |
| Yes | 601 (44.32) | 179 (43.98) | 422 (44.47) | |  | |  | |
| Drinking, n(%) |  |  |  | | 0.31 | | 0.575 | |
| No | 1,154 (85.10) | 343 (84.28) | 811 (85.46) | |  | |  | |
| Yes | 202 (14.90) | 64 (15.72) | 138 (14.54) | |  | |  | |
| Family history, n(%) |  |  |  | | 0.06 | | 0.809 | |
| No | 1,207 (89.01) | 361 (88.70) | 846 (89.15) | |  | |  | |
| Yes | 149 (10.99) | 46 (11.30) | 103 (10.85) | |  | |  | |
| Educational level, n(%) |  |  |  | | 1.92 | | 0.590 | |
| Illiterate | 267 (19.69) | 71 (17.44) | 196 (20.65) | |  | |  | |
| Primary school​ | 557 (41.08) | 170 (41.77) | 387 (40.78) | |  | |  | |
| Junior high school | 304 (22.42) | 95 (23.34) | 209 (22.02) | |  | |  | |
| High school or above | 228 (16.81) | 71 (17.44) | 157 (16.54) | |  | |  | |
| Occupational status, n(%) |  |  |  | | 1.81 | | 0.613 | |
| Retired​ | 303 (22.35) | 87 (21.38) | 216 (22.76) | |  | |  | |
| Employed​ | 158 (11.65) | 48 (11.79) | 110 (11.59) | |  | |  | |
| Unemployed​ | 871 (64.23) | 262 (64.37) | 609 (64.17) | |  | |  | |
| Other​ | 24 (1.77) | 10 (2.46) | 14 (1.48) | |  | |  | |
| Caregiver, n(%) |  |  |  | | 4.44 | | 0.109 | |
| Spouse | 532 (39.23) | 177 (43.49) | 355 (37.41) | |  | |  | |
| Children | 689 (50.81) | 193 (47.42) | 496 (52.27) | |  | |  | |
| Variables | Overall  (N = 1,356) | Training Set  (n = 949) | Validation Set  (n = 407) | | Z/χ² | | *P* | |
| Self | 135 (9.96) | 37 (9.09) | 98 (10.33) | |  | |  | |
| BI | 90.00  (50.00, 100.00) | 90.00  (50.00, 100.00) | 90.00  (50.00, 100.00) | | -0.27 | | 0.785 | |
| WBC, ×10⁹/L | 7.65 (6.33, 9.37) | 7.73 (6.39,9.40) | 7.63 (6.32, 9.36) | | -0.60 | | 0.548 | |
| RBC, ×10^12^/L | 4.45 (4.08, 4.83) | 4.48 (3.96,4.87) | 4.44 (4.12, 4.81) | | -0.36 | | 0.716 | |
| Hb, g/L | 132.00  (121.00, 143.00) | 132.00  (119.00, 144.50) | 132.00  (122.00, 143.00) | | -0.48 | | 0.630 | |
| PLT, ×10⁹/L | 230.50  (192.00, 271.25) | 226.00  (191.00,271.00) | 233.00  (192.00, 273.00) | | -1.37 | | 0.169 | |
| NEU, ×10⁹/L | 5.15 (4.00, 6.95) | 5.22 (4.12,6.88) | 5.11 (3.98, 6.97) | | -0.74 | | 0.457 | |
| LYM, ×10⁹/L | 1.73 (1.31, 2.28) | 1.73 (1.33,2.26) | 1.73 (1.31, 2.29) | | -0.05 | | 0.958 | |
| MONO, ×10⁹/L | 0.53 (0.41, 0.69) | 0.53 (0.42,0.68) | 0.52 (0.40, 0.70) | | -0.63 | | 0.527 | |
| TP, g/L | 65.48  (62.00, 69.38) | 65.91  (62.44, 70.06) | 65.39  (62.00, 69.00) | | -1.57 | | 0.115 | |
| Serum albumin, g/L | 38.39  (36.46, 40.68) | 38.65  (36.39, 40.67) | 38.31  (36.47, 40.68) | | -0.82 | | 0.411 | |
| Serum globulin, g/L | 27.20  (24.54, 30.12) | 27.39  (24.80, 30.90) | 27.14  (24.46, 29.88) | | -1.82 | | 0.068 | |
| ALT, U/L | 17.05  (12.83, 24.45) | 17.06  (12.45, 26.51) | 17.04  (12.92, 23.74) | | -0.69 | | 0.491 | |
| AST, U/L | 19.38  (15.90, 24.45) | 18.97  (15.69, 24.49) | 19.42  (16.04, 24.41) | | -0.45 | | 0.652 | |
| GLU, mmol/L | 5.57 (4.88, 7.47) | 5.62 (4.91,7.61) | 5.55 (4.85, 7.42) | | -0.58 | | 0.565 | |
| TC, mmol/L | 4.76 (3.98, 5.56) | 4.84 (3.96,5.66) | 4.75 (3.99, 5.51) | | -0.89 | | 0.375 | |
| TG, mmol/L | 1.11 (0.92, 1.36) | 1.09 (0.91,1.33) | 1.12 (0.92, 1.36) | | -1.24 | | 0.216 | |
| HDL, mmol/L | 2.57 (1.30, 3.36) | 2.57 (1.36,3.46) | 2.57 (1.28, 3.35) | | -0.48 | | 0.628 | |
| LDL, mmol/L | 1.60 (1.11, 2.58) | 1.62 (1.15,2.58) | 1.58 (1.09, 2.56) | | -0.89 | | 0.375 | |
| Hypertension, n(%) |  |  |  | | 0.41 | | 0.520 | |
| No | 288 (21.24) | 82 (20.15) | 206 (21.71) | |  | |  | |
| Yes | 1,068 (78.76) | 325 (79.85) | 743 (78.29) | |  | |  | |
| Diabetes Mellitus, n(%) |  |  |  | | 0.74 | | 0.391 | |
| No | 800 (59.00) | 233 (57.25) | 567 (59.75) | |  | |  | |
| Yes | 556 (41.00) | 174 (42.75) | 382 (40.25) | |  | |  | |
| Variables | Overall  (N = 1,356) | Training Set  (n = 949) | | Validation Set  (n = 407) | Z/χ² | *P* | |  |
| Atrial Fibrillation, n(%) |  |  | |  | 3.03 | 0.082 | |  |
| No | 1,288 (94.99) | 393 (96.56) | | 895 (94.31) |  |  | |  |
| Yes | 68 (5.01) | 14 (3.44) | | 54 (5.69) |  |  | |  |
| Hyperlipidemia, n(%) |  |  | |  | 0.63 | 0.426 | |  |
| No | 1,165 (85.91) | 345 (84.77) | | 820 (86.41) |  |  | |  |
| Yes | 191 (14.09) | 62 (15.23) | | 129 (13.59) |  |  | |  |
| Number of stroke episodes, n(%) |  |  | |  | 0.63 | 0.730 | |  |
| Once | 1,087 (80.16) | 324 (79.61) | | 763 (80.40) |  |  | |  |
| Twice | 227 (16.74) | 72 (17.69) | | 155 (16.33) |  |  | |  |
| ≥ 3 times | 42 (3.10) | 11 (2.70) | | 31 (3.27) |  |  | |  |
| NIHSS | 3.00 (1.00, 5.00) | 3.00 (1.00,5.00) | | 3.00 (1.00, 5.00) | -0.76 | 0.445 | |  |
| Data presented are median (Q1, Q3) or n (%).  BI, Barthel Index; WBC, white blood cell; RBC, red blood cell; Hb, hemoglobin; PLT, platelet; NEU, neutrophil; LYM, lymphocyte; MONO, monocyte; TP, total protein; ALT, alanine transaminase; AST, aspartate transaminase; GLU, glucose; TC, total cholesterol; TG, triglycerides; HDL, high density lipoprotein; LDL, low density lipoprotein; NIHSS, National Institutes of Health Stroke Scale. | | | | | | | |  |

Table S2 Training set and validation set confusion matrix

| Data | AUC  (95% CI) | Accuracy (95% CI) | Sensitivity (95% CI) | Specificity (95% CI) | PPV  (95% CI) | NPV  (95% CI) | cut off |
| --- | --- | --- | --- | --- | --- | --- | --- |
| Training Set | 0.90 (0.88-0.93) | 0.87 (0.85-0.89) | 0.89  (0.87-0.91) | 0.79  (0.74-0.85) | 0.94 (0.92-0.96) | 0.66 (0.60-0.72) | 0.273 |
| Validation Set | 0.85 (0.80-0.89) | 0.80 (0.76-0.84) | 0.84 (0.80-0.88) | 0.69  (0.60-0.78) | 0.89 (0.86-0.93) | 0.58 (0.49-0.67) | 0.273 |
| AUC, Area under the curve; PPV, positive predictive value; NPV, negative predictive value; CI Confidence Interval | | | | | | | |

**
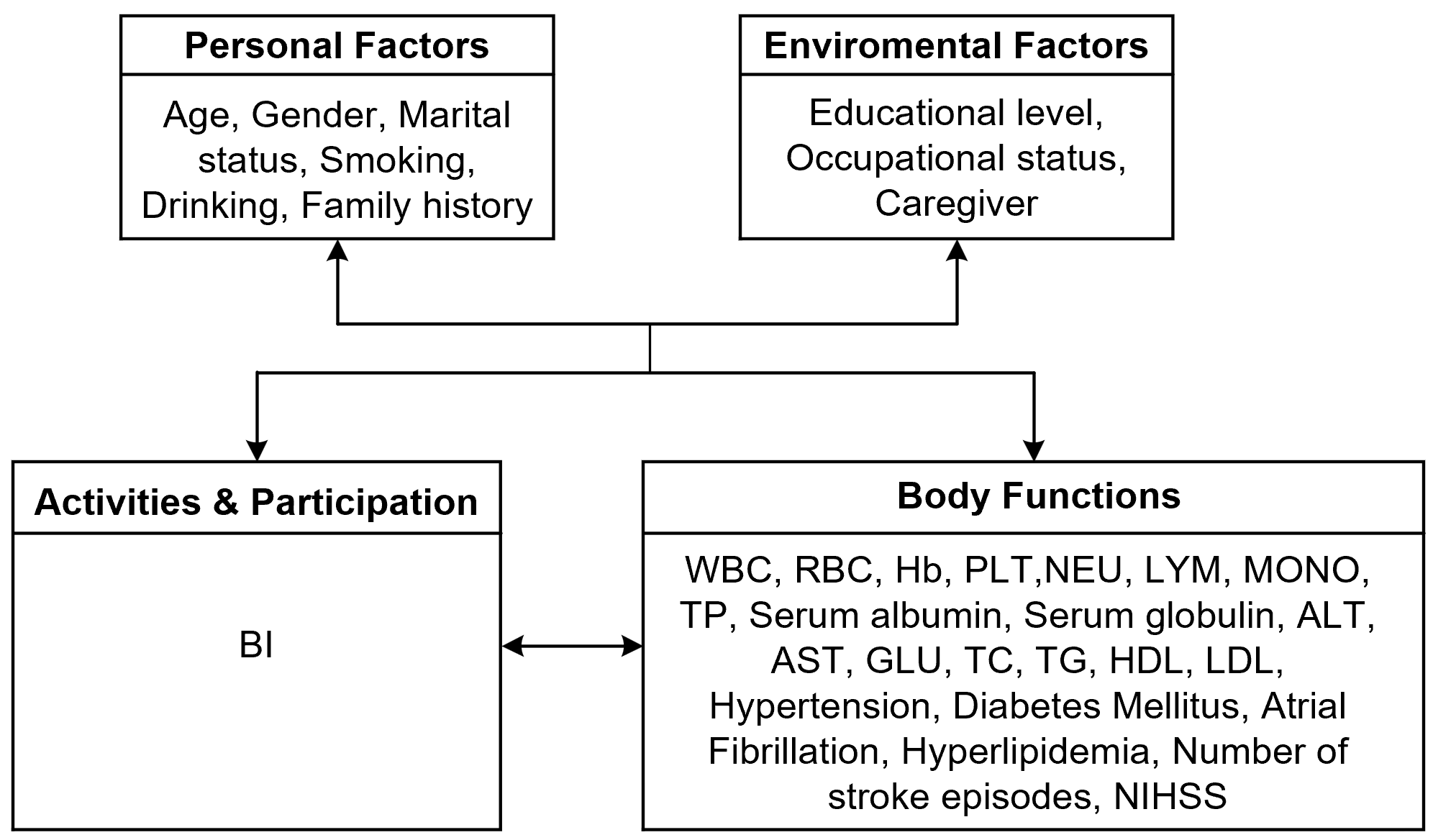
Fig. S1** Classification of predictive factors based on the ICF framework
